# Gender Dysphoria Preceding Intersex Recognition Demonstrates a Biophysiological Basis for Sex Identity

**DOI:** 10.21203/rs.3.rs-6623724/v1

**Published:** 2025-05-26

**Authors:** Beverly Rice, Billy Troy Wooton, Joshua G. Burkhart, Kartik Saini, Alexander J. Stokes, Helen Turner

**Affiliations:** Laboratory of Experimental Medicine, Department of Cell & Molecular Biology, John A. Burns School of Medicine, University of Hawaii at Manoa, Honolulu, HI, USA; United Nations CIFAL Center Honolulu, Chaminade University of Honolulu, Honolulu, HI, USA.; Laboratory of Experimental Medicine, Department of Cell & Molecular Biology, John A. Burns School of Medicine, University of Hawaii at Manoa, Honolulu, HI, USA; United Nations CIFAL Center Honolulu, Chaminade University of Honolulu, Honolulu, HI, USA.; Laboratory of Experimental Medicine, Department of Cell & Molecular Biology, John A. Burns School of Medicine, University of Hawaii at Manoa, Honolulu, HI, USA; Laboratory of Experimental Medicine, Department of Cell & Molecular Biology, John A. Burns School of Medicine, University of Hawaii at Manoa, Honolulu, HI, USA; Laboratory of Experimental Medicine, Department of Cell & Molecular Biology, John A. Burns School of Medicine, University of Hawaii at Manoa, Honolulu, HI, USA; United Nations CIFAL Center Honolulu, Chaminade University of Honolulu, Honolulu, HI, USA.; United Nations CIFAL Center Honolulu, Chaminade University of Honolulu, Honolulu, HI, USA.

**Keywords:** Gender Dysphoria (GD), Intersex, Prevalence, Electronic Health Records (EHR), Diagnostic Sequencing, Gender Identity, Health Disparities, Clinical Recognition, Biophysiological Diversity, Gender-Affirming Care, Policy Impact

## Abstract

Intersex individuals—those born with congenital variations in sex characteristics (VSCs)—experience elevated and underrecognized clinical risks. In an analysis of 3.42 million electronic health records, we identified 2,207 intersex patients, of whom 2.95% were diagnosed with gender dysphoria (GD)—nearly four times the rate in the general population (OR = 3.84, p < 0.001). In temporally resolvable cases, GD preceded intersex recognition in 47% of patients. Intersex individuals also received GD diagnoses at significantly older ages than non-intersex peers (median 32 vs. 26 years), with a broader and more variable age distribution. These patterns suggest delayed recognition, elevated psychosocial burden, and systemic misalignment between clinical frameworks and patient realities. Despite clinical recognition of both intersex traits and GD via long-established ICD codes, recent policies increasingly define sex as binary and reframe GD as a behavioral concern. Our findings suggest a biophysiological link between sex variation and gender identity distress, underscoring the need for care models that resist institutional erasure and systemic exclusion.

Intersex individuals, whose sex characteristics do not align neatly with male–female binaries, represent 1.7–2.3% of the global population—over 188 million people worldwide^1,2^. Despite this, their existence is often erased by policies that define sex strictly as male or female, excluding intersex people from healthcare systems, legal recognition, and scientific datasets. This erasure persists even as established diagnostic frameworks, including International Classification of Diseases (ICD) codes, formally recognize intersex traits. Such contradictions between clinical recognition and political or policy exclusion reflect longstanding tensions in research, medical practice, and public health—rooted not in science, but in stigma, ignorance, phobic attitudes, outdated ideals of sexual dimorphism, and systemic discomfort with sex and gender diversity.

Gender dysphoria (GD)—distress resulting from a mismatch between gender identity and assigned sex at birth—was nearly four times more common among intersex individuals than in the general population in our study. Despite this stark disparity, the intersection of GD and intersex traits remains critically understudied^3^. While GD has been extensively analyzed in non-intersex populations, little is known about its prevalence or clinical trajectory among people with intersex variations, which span chromosomal, hormonal, physiological, gonadal, and anatomical differences^4^. To address this gap, we analyzed diagnostic patterns in electronic health records (EHRs) from over 3.4 million patients, including 2,207 with intersex-related diagnoses. A significant almost fourfold higher prevalence of gender dysphoria was found among intersex individuals (2.95%) compared to non-intersex peers (0.78%), with an odds ratio of 3.84 (p < 0.001; Fig. 1A).

These findings underscore a disproportionate burden of GD among intersex individuals, warranting closer investigation into how intersex traits may shape gender identity experiences and clinical trajectories. To explore diagnostic patterns, we examined patients for both GD and intersex diagnoses.

Of these, 47.3% were diagnosed with GD before the intersex condition, 36.4% received the intersex diagnosis first, and 16.4% were diagnosed with both during the same clinical encounter (Fig. 1B).

A statistically significant and strong positive correlation was found between the year of gender dysphoria (GD) diagnosis and the year of intersex diagnosis (Spearman’s r = 0.77, p < 0.001; Fig. 1C), with a median gap of less than one year between the two. This temporal proximity indicates that the two conditions are frequently identified within the same clinical period, highlighting the importance of integrated, concurrent evaluation when either diagnosis is made.

Further, we found that intersex individuals are diagnosed with GD at significantly older ages than non-intersex individuals (Fig. 2). Non-intersex patients are typically diagnosed in early adulthood (mean = 28.2, median = 26, IQR = 21–32), whereas intersex patients exhibit a broader and older age distribution (mean = 36.2, median = 32, IQR = 27–47.5). Among those diagnosed with GD, individuals diagnosed after their intersex condition were older than those diagnosed beforehand, although both subgroups lagged behind the non-intersex cohort in age at diagnosis.

The elevated prevalence and delayed recognition of GD in intersex populations raise critical clinical and ethical concerns. Nearly half of those with both diagnoses were first identified with GD before their intersex condition, indicating that gender identity distress may precede or prompt deeper investigation into biological sex traits. These patterns challenge longstanding norms of irreversible gender assignment in infancy—procedures that risk long-term misalignment with an individual's affirmed identity. As the literature show, such "normalizing" interventions can have lasting psychological and physical consequences^6-9^

Stigma within healthcare settings continues to hinder timely and accurate diagnoses of intersex traits. Clinicians may avoid using the term “intersex” due to cultural discomfort, institutional norms, or fears of reinforcing stigma, often substituting vague or euphemistic labels that obscure the underlying diagnosis^6,10-11^. Some providers may even omit documentation of intersex traits entirely, motivated by concerns about discrimination or system-level pressures^12-13^. At a structural level, research and clinical infrastructures—including genomic databases and national cohorts—remain organized around binary definitions of sex, reinforcing invisibility and contributing to persistent data gaps, even in the presence of clinically coded intersex diagnoses.

These realities reinforce the need for integrated, multidisciplinary care teams that include endocrinologists, mental health providers, geneticists, and social workers. Such teams can better coordinate responses to complex cases involving both intersex traits and gender identity concerns. Policy changes are also essential: prohibiting non-consensual gender assignment surgeries in infancy and expanding access to psychosocial support throughout the life course are critical steps toward affirming care.

The research landscape for intersex health remains precarious. Despite promising early efforts to integrate intersex cohorts and codes into clinical research frameworks, mounting political backlash has reversed progress. Policies that reassert binary-only sex definitions are excluding intersex people from data collection, clinical trials, and research priorities. As we argue, the resulting "intersex research void" perpetuates misdiagnoses and systemic neglect^2,14-15^.

The erasure of intersex people from health systems and scientific discourse reflects a broader policy trend toward entrenched binary definitions of sex and gender. This erasure is not isolated. Transgender individuals increasingly face parallel forms of exclusion, particularly as legislative actions restrict gender-affirming care while paradoxically allowing irreversible surgeries on intersex infants^16-17^. The legal and medical systems often fail to recognize the biological complexity underpinning both groups—despite evidence from neuroimaging, endocrinology, and genetics that demonstrate brain–body, hormone–identity, and chromosomal–phenotypic incongruences in both intersex and transgender individuals^18-20^.

Some scholars have proposed rethinking sex classifications altogether, arguing that transgender identity may represent another point along a continuum of sex diversity—akin to intersex variation—grounded in biological and developmental science rather than purely social constructs^19,21-22^. These frameworks underscore that gender dysphoria should not be pathologized as a behavioral disturbance but recognized as a clinical response to biological incongruence—an understanding long reflected in ICD classifications^17^.

In this climate, clinicians should remain particularly attentive. Our findings show that nearly half of patients received a GD diagnosis before their intersex condition was recognized—suggesting that gender distress may serve as a gateway for uncovering deeper biological variance. Providers who encounter patients with GD might consider broader diagnostic evaluations, including chromosomal or hormonal testing, particularly when care options for transgender individuals are being restricted. Timely recognition of underlying intersex traits may not only support more accurate diagnosis and holistic care, but may also provide a measure of legal or clinical protection for patients whose identities are under threat in an increasingly politicized healthcare landscape^16,21^.

## Methods - online

### Data Source

This study utilized EHR data from the OCHIN-led Accelerating Data Value Across a National Community Health Center (ADVANCE) Clinical Research Network (CRN), a federally funded initiative supported by the Patient-Centered Outcomes Research Institute (PCORI). The ADVANCE CRN integrates outpatient EHR data from safety-net and federally qualified health center (FQHC) clinics, encompassing hospital, health plan, and community health data^22^. The dataset includes detailed patient-level information on demographics, clinical encounters, prescriptions, diagnoses, and vital signs. For this study, patient records collected since January 1, 2010, were analyzed, with inclusion restricted to individuals who met the study’s eligibility criteria.

### Data Curation and Standardization

Patient records were systematically consolidated to create comprehensive profiles, ensuring that all relevant conditions and diagnoses were captured. Diagnoses were standardized to the ICD-10^23^ format, with ICD-9 codes converted where necessary. Any non-convertible ICD-9 codes were excluded to maintain data integrity and consistency across records.

### Inclusion and Exclusion Criteria

Intersex patients were identified using ICD-10 codes associated with differences in sex characteristics, including chromosomal, gonadal, anatomical, and endocrine variations. The following diagnostic codes were used: Q50.02, Q51.0, Q51.811, Q51.5, Q51.821, Q52.0, Q54.8, Q55.0, Q55.1, Q55.5, Q55.62, Q56.0, Q56.1, Q56.2, Q56.3, Q56.4, Q96.0, Q96.1, Q96.2, Q96.3, Q96.4, Q96.8, Q96.9, Q97.0, Q97.1, Q97.2, Q97.3, Q97.4, Q97.8, Q97.9, Q98.0, Q98.1, Q98.3, Q98.4, Q98.5, Q98.6, Q98.7, Q98.8, Q98.9, Q99.0, Q99.1, E25, E25.0, E25.8, E25.9, E34.5, E34.50, E34.51, and E34.52. GD diagnoses were identified using ICD-10 codes F64.0, F64.1, F64.2, F64.8, and F64.9.

Patients were excluded from temporal analysis if both GD and intersex diagnoses were recorded during the first documented clinical encounter. In such cases, it was unclear whether the diagnoses were newly established or carried over from prior healthcare providers, meaning the actual timing of diagnosis could not be accurately determined. These records may have reflected historical diagnoses made at any point before the patient entered the system, rather than representing a true sequence of diagnostic events. Following the application of inclusion and exclusion criteria, a total of 55 patients met the criteria for inclusion in the temporal analysis.

### Statistical Analysis

Descriptive statistics were used to summarize patient characteristics, including age at diagnosis and diagnostic timing. The prevalence of GD among intersex individuals was compared to the non-Intersex population using Fisher’s Exact Test, which is appropriate for categorical data when expected cell counts are small. This test was selected over chi-square analysis due to its greater accuracy in small-sample scenarios. Odds ratios (OR) were calculated to quantify the strength of the association, providing a measure of the increased likelihood of GD diagnosis among intersex individuals relative to the general population.

To examine temporal relationships and differences in age at GD diagnosis, the following statistical techniques were employed based on the characteristics of the data:

- Spearman’s Correlation was used to assess the relationship between the years of GD and intersex diagnoses. This nonparametric test was selected because it evaluates monotonic relationships rather than assuming a linear relationship, making it robust against non-normally distributed data and potential outliers in diagnostic timing.
- Mann-Whitney U Tests were conducted to evaluate differences in:
  - Age at GD diagnosis between non-intersex (Case) and intersex individuals. The Mann-Whitney U test was selected due to the distribution of age data and the presence of outliers, ensuring that comparisons were not influenced by normality violations.
  - Age differences in GD diagnosis timing among intersex individuals, comparing those diagnosed with GD before versus after their intersex diagnosis to determine whether diagnostic sequence influenced age at onset. As with prior comparisons, the Mann-Whitney U test was used due to the nonparametric nature of the data.

All analyses were conducted using Python, with results validated against statistical software to ensure accuracy. Boxplots were generated to visually depict differences in age at GD diagnosis and the distribution of diagnostic sequencing among study participants, providing an intuitive representation of central tendencies and variability in diagnostic patterns.

## Study strengths and limitations

This study leverages a large, multi-state EHR dataset comprising over 3.4 million patients, including records from federally qualified health centers (FQHCs), offering rare insight into gender dysphoria (GD) and intersex diagnoses in a diverse, often underrepresented population. While robust, this real-world dataset carries limitations common to EHR-based research, including potential underreporting or misclassification due to inconsistent coding practices and reliance on ICD codes from 2010 onward. Diagnostic timing may be imprecise in cases with concurrent entries, and the relatively small number of intersex patients with GD limits subgroup analysis. Additionally, the observational nature of the study precludes causal inference, and unmeasured factors such as healthcare access and provider behavior may confound results. Despite these constraints, our findings offer valuable population-level evidence and highlight the need for greater diagnostic awareness, inclusive care models, and equity-driven policy reform.

## Figures and Tables

**Figure 1:**
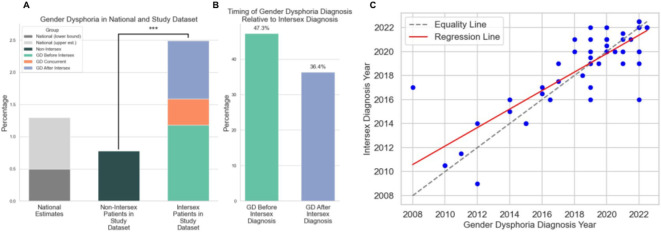
A) Prevalence and diagnostic breakdown of gender dysphoria (GD) in intersex and non-intersex populations. A bar plot comparing GD diagnosis prevalence in national estimates (0.5-1.3%)^5^, non-intersex (n ≈ 3.42 million) versus intersex patients (n = 2,207). The intersex group shows a significantly higher GD prevalence (2.95%) relative to non-intersex individuals (0.78%; odds ratio = 3.84, p = 9.26 × 10^−19^). The intersex bar is subdivided into three diagnostic categories: GD diagnosed before intersex, GD diagnosed after intersex, and GD and intersex diagnosed concurrently. An adjacent normalized bar removes the concurrent cases to emphasize directional diagnosis trends. B) Timing of gender dysphoria diagnosis relative to intersex diagnosis. A bar graph displays the percentage of intersex patients with GD diagnosed either before (47.3%) or after (36.4%) their intersex diagnosis. C) Correlation between year of Gender Dysphoria (GD) and intersex diagnoses. The scatter plot shows the relationship between the year of GD diagnosis (x-axis) and the year of intersex diagnosis (y-axis) for 46 patients. Each blue dot represents a single patient. The dashed gray line indicates the line of equality, where GD and intersex diagnoses occur in the same year, while the red regression line shows the linear trend between the two variables. A strong positive correlation is observed (Spearman’s r = 0.77, p < 0.001), suggesting that GD and intersex diagnoses are often made in close temporal proximity.

**Figure 2:**
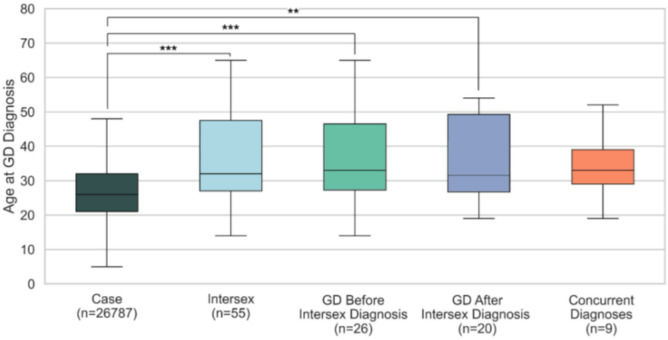
Age at gender dysphoria (GD) diagnosis across intersex and non-intersex groups. Boxplots depict the distribution of age at first GD diagnosis in five cohorts: non-intersex controls (n = 26,787), intersex individuals overall (n = 55), intersex individuals diagnosed with GD before their intersex condition (n = 26), intersex individuals diagnosed with GD after their intersex condition (n = 20), and individuals diagnosed with GD and their intersex condition concurrently (n = 9). Mann-Whitney U tests confirmed significant differences in the age at GD diagnosis between the Case and Intersex groups (U = 450,000.5, p = 5.84 × 10^−7^), and between Case and both the GD-before (p = 1.97 × 10^−4^) and GD-after subgroups (p = 4.03 × 10^−3^).
